# Molecular characterization of antimicrobial multi-drug resistance in non-typhoidal *Salmonellae* from chicken and clam in Mangalore, India

**DOI:** 10.7555/JBR.31.20160094

**Published:** 2017-05-28

**Authors:** Yemisi Olukemi Adesiji, Santhosh Kogaluru Shivakumaraswamy, Vijaya Kumar Deekshit, Girisha Shivani Kallappa, Indrani Karunasagar

**Affiliations:** 1. Department of Medical Microbiology and Parasitology, Ladoke Akintola University of Technology College of Health Sciences, Osogbo, Osun State 230222, Nigeria; 2. Nitte University Centre for Science Education and Research, UNESCO MIRCEN for Medical & Marine Biotechnology, NITTE University, Deralakatte, Mangalore 575018, India; 3. Department of Fisheries Microbiology, Karnataka Veterinary, Animal and Fisheries Sciences University, College of Fisheries Mangalore, Karnataka 575002, India.

**Keywords:** mutation, multi-drug resistant, non-typhoidal *Salmonellae*, plasmid mediated quinolone resistance, quinolone resistance determining region

## Abstract

*Salmonella enterica* has been documented as one of the leading causes of salmonellosis throughout the world and is most commonly associated with the consumption of contaminated food products. Thus, this research was aimed at studying the antimicrobial susceptibility pattern and detection of quinolone resistance in *Salmonella* spp isolated from food of animal origin. Thirty-six *Salmonella* isolates comprising 8 from poultry and 28 from seafood (clams) were identified, serotyped and characterized for their antimicrobial susceptibility against 10 different antibiotics. Plasmid DNA was isolated from all the isolates by alkaline lysis, quinolone resistant non-typhoidal *S. *Weltevreden were examined for mutation in the DNA gyrase coding gene. Among the 36 *Salmonella* isolates, 20 were *S. weltevreden* (8 from poultry and 12 from seafood) and 16 were *S. *Typhimurium (from seafood). All the isolates showed multiple resistance to nalidixic acid, tetracycline, co-trimoxazole and nitrofurantoin, but, interestingly, the isolates were 100% susceptible to ampicillin, chloramphenicol and gentamicin. Resistant isolates from the study carried the genes responsible for resistance to respective antibiotics. The strain S130 isolated in the study showed single point mutation, Asp87Gly, at position 87 in quinolone resistance determining region. It revealed mutation in quinolone resistance determining region as a cause for quinolone resistance in non-typhoidal *Salmonellae*. The occurrence of genes accountable for plasmid mediated resistance to quinolones (viz., *qnrA*, *qnrB* and *qnrS*) in plasmid of non-typhoidal *Salmonellae* isolates provides evidence for plasmid mediated quinolone resistance.

## Introduction

Non-typhoidal *Salmonellae* (NTS) has emerged as a major group of foodborne pathogens which causes gastroenteritis, septicaemia, endocarditis and subsequent infections like intra-abdominal infections, and pulmonary infections^[1]^. NTS have a wide range of hosts and are strongly associated with animals and agricultural products^[2]^. Hence salmonellosis can easily occur or spread by consumption of contaminated foods of animal origin, such as those from poultry, fish, eggs, beef and dairy products^[3]^. Both the developed and developing countries have recently reported continuous increase in the incidence of human salmonellosis in patients who are immunosuppressed, or at the extremes of age^[4]^. Traditionally, recommended regimens for the treatment for salmonellosis include the traditional first-line drugs like ampicillin, chloramphenicol and trimethoprim-sulfamethoxazole^[5]^. But emerging drug resistance over the past 25 years has limited the usefulness of these antimicrobial agents. Currently, quinolone and third-generation cephalosporin antibiotics are preferred for the treatment against salmonellosis^[5]^.

Quinolones and fluoroquinolones are an important class of broad spectrum antibiotics which possesses strong antimicrobial activity against many human pathogenic bacteria. They are usually used to treat urinary tract infections (UTI) and gastrointestinal infections^[6]^. This group of antibiotics mainly act on the bacterial DNA gyrase and topoisomerase IV, which are important in DNA replication^[7]^. 

In last recent years, resistance to quinolones and fluoroquinolones in NTS has been increasingly reported among human and veterinary isolates^[1]^. In the developing countries, this may be partly related to lack of legislation on the use of antibiotics. Bacteria may acquire resistance to quinolones *via* mutations in the *gyrA*, *gyrB*, *parC* and *parE* genes^[11^–^12]^. All mutations described reside within the quinolone resistance determining region of the A subunit of DNA gyrase, i.e. amino acids 67–122 and for some of the isolates the mechanism of resistance has been studied. In addition to mutation based resistance, Martinez *et al*.^[13]^ found quinolone resistance genes (*qnrA, qnrB* and *qnrS*) in plasmid and confirmed that resistance can also occur *via* plasmid mediated quinolone resistance.

Globally, the impact of NTS on human health is very high and earlier reports have documented the emergence of multidrug resistant *Salmonella* in developing and developed countries^[5^,^14^–^16]^. The globalization of the food trade has increased the likelihood of international incidents of food contamination through travels, migration and distribution of food^[14]^. This increases the wide spread of resistance determinants in bacteria associated with food and even in clinical pathogens. The analysis of food samples from animal origin could provide useful information on resistance pattern of *Salmonella* against different antimicrobial agents. Hence, the present study attempted to determine antibiotic susceptibility and possible causes of resistance to quinolones in NTS strains isolated from food of animal origin.

## Materials and methods

### Isolation of bacteria and antibiogram analysis

Mollusc (clam) samples (132) from the fish market and landing centers in Mangalore and also chicken samples (78) from the poultry and chicken market outlets in Mangalore (***Table 1***) were collected aseptically. Samples were brought to the laboratory and immediately processed for analysis. *Salmonella* strains were isolated from the samples by culture-based conventional method as recommended by the FDA Bacteriological Analytical Manual^[18]^ and identified using standard biochemical tests as previously described^[19]^. Pre-enriched samples (10 g of chicken or clam meat) in lactose broth (90 mL) were enriched in selenite cysteine broth (SCB) and streaked in selective xylose lysine deoxy-cholate agar (XLD) plate. Typical colonies from each of the selective plates were sub-cultured on to Luria Bertani agar and subjected to a battery of biochemical tests for identification of *Salmonella*. The biochemically identified isolates were further confirmed by polymerase chain reaction (PCR) with *invA* and *hns* gene primers (***Table 2***). PCR confirmed isolates were then serotyped at the National *Salmonella* and *Escherichia* Centre, Central Research Institute, Kasauli, India. Antibiotic susceptibility test was performed for all the isolates by disk diffusion method using standard procedure^[26]^ on Mueller-Hinton agar as recommended by the Clinical and Laboratory Standards Institute. The antibiotics included in the test were nalidixic acid (30 mcg), tetracycline (30 mcg), co-trimoxazole (25 mcg), ciprofloxacin (5 mcg), chloramphenicol (30 mcg), ampicillin (10 mcg), gentamicin (10 mcg), nitrofurantoin (300 mcg), imipenem (10 mcg) and piperacillin-tazobactam (100/10 mcg). Minimum inhibitory concentration (MIC) was performed using Ezy MIC^TM^ strips (HiMedia Laboratories Pvt. Ltd., India) for nalidixic acid and ciprofloxacin. Multiple antibiotic resistance (MAR) index for each resistance pattern was calculated by using the formula by Singh *et al*.^[27]^.

**Tab.1:** Occurrence and distribution of ***Salmonella*** in seafood (clams) and poultry samples.

Si No.	Source	No. of sampling	No. of positive samples	Percentage of positive samples	Serotypes (No.)
1	Seafood	132	28	21.2	*S.* Weltevreden (12) *S. *Typhimurium (16)
2	Poultry	78	8	10.2	*S.* Weltevreden (8)
	Total	210	36	17.1	

**Tab.2:** List of primers used for  detection of antibiotic resistant determinants in the study.

Target	Gene	Sequence 5′- 3′	Size	Tm (°C)	Reference
Salmonella	invA	GTGAAATTATCGCCACGTTCGGGCAA	284 bp	64	Rahn *et al*.^[20]^
		TCATCGCACCGTCAAAGGAACC			
	hns	TACCAAAGCTAAACGCGCAGCT	156 bp	60	Jones *et al*.^[21]^
		TGATCAGGAAATCTTCCAGTTGC			
Gyrase	gyrA	CTGAAGCCGGTACACCGTCG	290 bp	55	Casin *et al*.^[22]^
		TCGGCCATCAGTTCGTGGGC			
Quinolone resistance protein	qnrA	ATTTCTCACGCCAGGATTTG	516 bp		Robicsek *et al*.^[23]^
		GATCGGCAAAGGTTAGGTCA			
	qnrB	GATCGTGAAAGCCAGAAAGG	469 bp		
		ACGATGCCTGGTAGTTGTCC			
	qnrS	ACGACATTCGTCAACTGCAA	417 bp		
		TAAATTGGCACCCTGTAGGC			
Antibiotic resistant genes	tetA	TTGGCATTCTGCATTCACTC	494 bp		Menggen *et al*.^[24]^
		GTATAGCTTGCCGGAAGTCG			
	tetB	CAGTGCTGTTGTTGTCATTAA	571 bp		
		GCTTGGAATACTGAGTGTTAA			
	tetC	CTTGAGAGCCTTCAACCCAG	418 bp		
		ATGGTCGTCATCTACCTGCC			
	tetS	TGGAACGCCAGAGAGGTATT	660 bp		Aarestrup *et al*.^[25]^
		ACATAGACAAGCCGTTGACC			
	Sul1	TTTCCTGACCCTGCGCTCTAT	425 bp		Menggen *et al*.^[24]^
		GTGCGGACGTAGTCAGCGCCA			
	Sul2	CCTGTTTCGTCCGACACAGA	435 bp		
		GAAGCGCAGCCGCAATTCAT			
	Sul3	ATGAGCAAGATTTTTGGAATCGTAA	792 bp		
		CTAACCTAGGGCTTTGGATATTT			

### Analysis of antibiotic resistance determinants by PCR

Genomic DNA was extracted from the bacterial culture by CTAB (Cetyl trimethyl ammonium bromide) method as described by Ausubel *et al*.^[28]^. All the resistant isolates were analyzed for the presence of specific genes encoding for resistance to particular antibiotics using the primers listed in the ***Table 2***. PCR was carried out in 30 µL reaction mixture containing 10X buffer (100 mmol/L Tris-HCl, pH 8.3, 20 mmol/L MgCl_2_, 500 mmol/L KCl, 0.1% gelatin), 200 mmol/L of dNTPs (dATP, dTTP, dGTP, dCTP), 10 pmol each of forward and reverse primers and 1.0 unit of Taq DNA polymerase enzyme with 2 µL of template DNA. Amplification was carried out in a MJ-Research Thermo Cycler (PTC-200, USA) with the optimized PCR program consisted of an initial denaturation at 94°C for 5 minutes, followed by 35 cycles with each cycle consisting of 94°C for 60 seconds, annealing temperature of X °C (based on primer used) for 60 seconds and extension at 72°C for 30 seconds. The final extension was set to 72°C for 10 minutes.

### Detection of mutation in ***gyrA*** gene from quinolone resistant isolates

Bacterial strains resistant to quinolone (nalidixic acid/ ciprofloxacin) were selected and subjected to mutation study at the gyraseA gene. The *gyrA* gene was amplified by PCR using oligonucleotide primers (***Table 1***) with extracted genomic DNA (as described above), under specified cyclic conditions. Amplified PCR product was purified using a QIAquick PCR purification Kit (Qiagen, Hilden, Germany) and sequenced by Bio serve Biotechnologies (INDIA) Pvt. Ltd, Hyderabad, India. The obtained nucleotide sequences were analyzed using blast programs (http://blast.ncbi.nlm.nih.gov/Blast.cgi). The sequences were aligned and compared with the sequence of the *gyrA* gene of quinolone sensitive strain reported in the GenBank (Accession no. EU898544) by using multalign program (http://multalin.toulouse.inra.fr/multalin/multalin.htm). The amino acids were deduced from the DNA sequences obtained through a Molecular Tool Kit web program (http://www.vivo.colostate.edu/molkit). The nucleotide sequences were then submitted to GenBank.

### Evaluation of Plasmid mediated quinolone resistance

Plasmid DNA was isolated from all the isolates by alkaline lysis method described by Sambrook *et al*.^[29]^. Isolated plasmid DNA was profiled using agarose gel electrophoresis in 1X Tris-acetate-EDTA buffer on 0.8% agarose gel. The separated bands were extracted by using gel extraction kit (Chromous Biotech, Bangalore, India). Extracted plasmid DNA was used as template to check quinolone resistance genes (*qnrA, qnrB* and *qnrS*) located in plasmid or chromosome, using the oligonucleotide primers (***Table 2***) by PCR.

### Statistical analysis

Descriptive statistics was used to describe frequency and percentages of *Salmonella* positive isolates from food samples.

## Results

### Identification of bacterial strain and antimicrobial susceptibility tests

**Fig.1:**
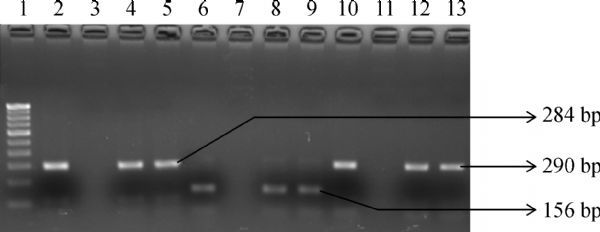
Gel-electrophoresis of PCR amplified products of ***Salmonella ***isolates and their ***gyrA*** gene. Lane1: 100 bp DNA ladder; Lane 2: invA gene positive control (284 bp); Lane 3: invA gene negative control; Lane 4 and 5: Positive *Salmonella* isolates (positive *invA* gene); Lane 6: Positive control for *hns* gene (156 bp); Lane 7: Negative control for *hns* gene; Lane 8 and 9: Positive *Salmonella* isolates (positive *hns* gene); Lane 10: Positive control for *gyrA* gene (290 bp); Lane 11: Negative control for *gyrA* gene; Lane 12 to 13: *gyrA* gene of isolates

All the 36 strains isolated from different sources in the study were identified as *Salmonella* spp. by conventional methods and confirmed by PCR for *invA* and *hns* gene by yielding a product of 284 bp and 156 bp, respectively (***Fig. 1***). Of total 36 isolates, eight strains were isolated from 78 poultry samples and 28 were isolated from 132 seafood (clams) samples (***Table 2***). Confirmed isolates were serotyped and subjected to further studies. Among 36 *Salmonella* isolates, 20 were *S.* Weltevreden (8 from poultry and 12 from seafood) and 16 were *S. *Typhimurium (from seafood). All 36 isolates (100%) examined in the study were multi-drug resistant (MDR) i.e., resistant to more than two antibiotics and showed MAR index ranging from 0.40-0.50 (***Table 3***). ***Table 3*** further shows the antibiogram of the results which includes the isolates/sources and their respective resistant gene to the tested antibiotics.

They showed resistance to nalidixic acid, tetracycline and co-trimoxazole. Twenty two (61%) isolates showed resistance to nitrofurantoin, 16 (44%) were resistant to piperacillin-tazobactam and four (11%) were resistant to imipenem. But surprisingly all the isolates were susceptible to ampicillin, chloramphenicol, ciprofloxacin and gentamicin. Isolates resistant to nalidixic acid showed MIC of more than 256 µg/mL and the MIC of isolates for ciprofloxacin was ranged between 0.032 to 1.00 µg/mL.

**Fig.2:**
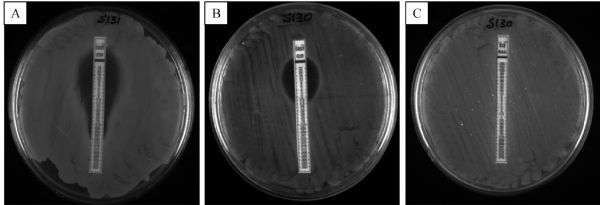
Multialigned sequences of gyrA gene of S130 (present study) and TW14359 (from GenBank). Highlight (oval shape) indicates the change in nucleotide sequence at 259 from adenine (A) to guanine (G) which leads to change in amino acid at position 87 (in QRDR region).

### PCR analysis for antibiotic resistant genes

The isolates were tested for presence of antibiotic resistance genes viz. *tetA*, *tetB*, *tetC*, and *tetS* for tetracycline and *sul1*, *sul2*, *sul3* for sulphonamides. All (100%) tetracycline resistant isolates amplified 494 bp of PCR product with *tetA* gene, 32 (89%) of them amplified 660 bp of PCR product with *tetS* gene, but none of them tested positive for *tetB* and *tetC*. Among the isolates resistant to co-trimoxazole, eight (23%) yielded 425 bp PCR product when amplified with *sul1* gene primer, nevertheless none of the resistant isolates showed positive for *sul2* and *sul3* gene.

**Tab.3:** Antibiotic sensitivity/resistance pattern of ***Salmonella*** isolates.

Strain	Source	Antibiotic resistant pattern-Phenotype	*Salmonella* Serovar	Antibiotic resistant pattern-Genotype	MAR index
S130	Poultry	NA,TE,COT,NIT,IPM,	*S*. Weltevreden	TetA,Sul1, TetS, qnrA, qnrS	0.50
S131	Poultry	NA,TE,COT,NIT,	*S*. Weltevreden	TetA,Sul1, qnrA, qnrS	0.40
S132	Seafood	NA,TE,COT,NIT,PIT,	*S*. Weltevreden	TetA, TetS, qnrB, qnrS	0.50
S133	Seafood	NA,TE,COT,NIT,PIT,	*S*. Weltevreden	TetA, TetS, qnrA, qnrS	0.50
S188	Seafood	NA,TE,COT,NIT,	*S*. Typhimurium	TetA, TetS, qnrB, qnrS	0.40
S189	Seafood	NA,TE,COT,NIT,PIT,	*S*. Weltevreden	TetA, TetS,	0.50
S190	Seafood	NA,TE,COT,NIT,PIT,	*S*. Weltevreden	TetA, TetS,	0.50
S191	Seafood	NA,TE,COT,NIT,PIT,	*S*. Weltevreden	TetA, TetS, qnrA, qnrS	0.50
S192	Seafood	NA,TE,COT,NIT,	*S*. Typhimurium	TetA, TetS,	0.40
S193	Seafood	NA,TE,COT,NIT,	*S*. Typhimurium	TetA, TetS, qnrB, qnrS	0.40
S194	Seafood	NA,TE,COT,NIT,PIT,	*S*. Typhimurium	TetA, TetS, qnrB, qnrS	0.50
S195	Seafood	NA,TE,COT,NIT,PIT,	*S*. Weltevreden	TetA, TetS, qnrA. qnrS	0.50
S210	Poultry	NA,TE,COT,NIT,IPM,	*S*. Weltevreden	TetA,Sul1, TetS, qnrA, qnrS	0.50
S211	Seafood	NA,TE,COT,NIT,PIT,	*S*. Weltevreden	TetA, TetS,	0.50
S222	Seafood	NA,TE,COT,NIT,	*S*. Typhimurium	TetA, TetS,	0.40
S223	Seafood	NA,TE,COT,NIT,PIT,	*S*. Typhimurium	TetA, TetS, qnrB, qnrS	0.50
S224	Seafood	NA,TE,COT,NIT,PIT,	*S*. Weltevreden	TetA, TetS, qnrA, qnrS	0.50
S225	Seafood	NA,TE,COT,NIT,PIT,	*S*. Weltevreden	TetA, TetS, qnrB, qnrS	0.50
S226	Poultry	NA,TE,COT,NIT,	*S*. Weltevreden	TetA,Sul1, qnrA, qnrS	0.40
S227	Seafood	NA,TE,COT,NIT,	*S*. Typhimurium	TetA, TetS, qnrB, qnrS	0.40
S228	Poultry	NA,TE,COT,NIT,IPM,	*S*. Weltevreden	TetA,Sul1, TetS, qnrA, qnrS	0.50
S229	Seafood	NA,TE,COT,NIT,PIT,	*S*. Typhimurium	TetA, TetS,	0.50
S230	Seafood	NA,TE,COT,NIT,PIT,	*S*. Weltevreden	TetA, TetS, qnrA, qnrS	0.50
S278	Seafood	NA,TE,COT,NIT,	*S*. Typhimurium	TetA, TetS,	0.40
S279	Poultry	NA,TE,COT,NIT,	*S*. Weltevreden	TetA,Sul1, qnrA, qnrS	0.40
S280	Seafood	NA,TE,COT,NIT,	*S*. Typhimurium	TetA, TetS, qnrB, qnrS	0.40
S281	Seafood	NA,TE,COT,NIT,	*S*. Typhimurium	TetA, TetS,	0.40
S282	Seafood	NA,TE,COT,NIT,PIT,	*S*. Typhimurium	TetA, TetS,	0.50
S283	Seafood	NA,TE,COT,NIT,PIT,	*S*. Weltevreden	TetA, TetS, qnrA, qnrS	0.50
S284	Seafood	NA,TE,COT,NIT,	*S*. Typhimurium	TetA, TetS, qnrB, qnrS	0.40
S296	Seafood	NA,TE,COT,NIT,	*S*. Typhimurium	TetA, TetS, qnrB, qnrS	0.40
S297	Poultry	NA,TE,COT,NIT,IPM,	*S*. Weltevreden	TetA,Sul1, TetS, qnrA, qnrS	0.50
S298	Seafood	NA,TE,COT,NIT,	*S*. Typhimurium	TetA, TetS,	0.40
S299	Poultry	NA,TE,COT,NIT,	*S*. Weltevreden	TetA,Sul1, qnrA, qnrS	0.40
S300	Seafood	NA,TE,COT,NIT,	*S*. Typhimurium	TetA, TetS, qnrB, qnrS	0.40
S301	Seafood	NA,TE,COT,NIT,PIT,	*S*. Weltevreden	TetA, TetS, qnrA, qnrS	0.50

NA: nalidixic acid, TE: tetracycline, COT: co-trimoxazole, NIT: nitrofurantoin, PIT: piperacillin-tazobactam, IPM: imipenem. MAR index= Number of resistance antibiotics/total number of antibiotics tested.

### Analysis of ***gyrA*** gene sequence of quinolone resistant isolates

**Fig.3:**
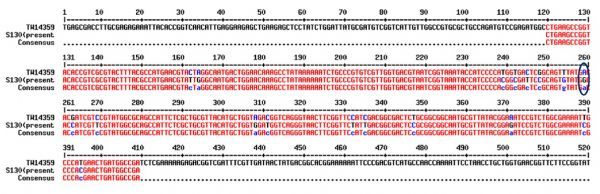
Partial amino acid sequence of gyraseA gene of strain TW14359 and isolate S130. Amino acid in the sequences at the position 87 (highlighted) attesting QRDR region indicates point mutation Asp87Gly.

Nalidixic acid resistant isolates were selected and studied for detection of mutation by using *gyrA* primers, since it has been reported that a point mutation in the *gyrA* gene coding for the A subunit of gyrase could cause resistance for quinolone. PCR amplification with *gyrA* primer yielded 290 bp product (***Fig. 1***) and the product was purified and sequenced. The obtained nucleotide sequences were compared with other *gyrA* gene sequence of quinolone sensitive strain. In the *gyrA* sequence of isolate S130, transition of nucleotide from guanine to adenine (G→A) at 259^th^ position in the quinolone resistance determining region was observed (***Fig. 2***). This point mutation in *gyrA* sequence resulted in the replacement of aspartic acid by glycine (asp-87-gly) at position 87 (***Fig. 3***). But the *gyrA* sequence of other isolates was similar with the sensitive strain sequence. Sequence of S130 was deposited at GenBank under the accession number KC570463. Of interest, the *gyrA* sequence of S130 also showed change in nucleotide (point mutation) at other different points but that mutation did not result change in amino acid.

**Fig.4:**
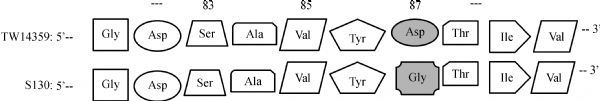
Detection of PCR amplified products of quinolone resistance protein coding genes. Lane 1:100 bp DNA ladder, Lane 3 to 5: qnrA (516 bp), Lane 7 to 9: qnrB (469 bp), Lane 11 to 13: qnrS (417 bp) and Lanes 2, 6, 10 negative controls.

### Plasmid mediated quinolone resistance

Apart from the mutations in the chromosomal gene of quinolone resistant isolates, the plasmid mediated quinolone resistance was tested for the presence of qnr genes (*qnrA*, *qnrB* and *qnrS*). Total 26 (72%) isolates were tested positive for any of the three tested *qnr* genes. Out of the 36 isolates, 15 (42%) tested positive for *qnrA* gene by yielding 516 bp of PCR product, 11 (30%) amplified 469 bp PCR product of *qnrB* gene and 26 (72%) isolates amplified 417 bp PCR product of *qnrS* (***Fig. 4***).

## Discussion

Previously, non-typhoidal *Salmonella* (NTS) infections were commonly treated with ampicillin, chloramphenicol and trimethoprim-sulfamethoxazole. With the emergence of resistance to those first line antibiotics quinolones, fluoroquinolones and third-generation cephalosporins are used for treatment^[30]^. But their widespread use has developed a selective pressure on NTS and triggered resistance against them^[2]^. Earlier reports from different developing countries revealed high multi-drug resistance rate in NTS isolated from humans and poultry^[2]^. Correspondingly, data from the present study harbingers the acquirement of multi-drug resistance (MDR) in NTS isolated from the poultry and seafood products. All 36 isolates (both from poultry and seafood) showed resistance to at least more than three antibiotics tested in the study. The isolates showed 100% resistance to nalidixic acid, tetracycline, co-trimoxazole and nitrofurantoin. Of 36 isolates, 16 (44%) showed resistance to piperacillin-tazobactam and 4 (11%) were resistant to imipenem.

Of late many researchers observed that due to low level use of first line antibiotics, they have re-emerged and are used to treat multi-drug bacterial isolates^[31]^. Snehali and Mohammed^[32]^ reported that the older antibiotics such as polymyxins, co-trimoxazole, aminoglycosides and chloramphenicol are more active and can be used to treat many of the MDR, XDR and PDR strains. However, interestingly in our study all the isolates (100%) were susceptible to ampicillin, chloramphenicol and gentamicin. It prominently demonstrates that these antibiotic compounds can be effectively used to treat against multi-drug NTS infections. But currently reason remains unclear and therefore further studies need to be carried out with NTS isolated from the study against these compounds.

Quinolones were highly recommended and was a first choice for treatment of serious intestinal *Salmonella* infections in adults but development of resistance reduced the efficacy of provisional treatment^[33]^. Acquisition of this desperate resistance to quinolones has been developed due to point mutations in chromosome at quinolone resistance-determining region (QRDR) of DNA gyrase and topoisomerase IV gene and along with plasmid mediated resistance mechanisms^[5]^. Change in a single nucleotide (mutation) in *gyraseA* gene sequence, which resulted in change in confirmation of protein (DNA gyrase) will prevent binding of antimicrobial agents and carrying out antimicrobial activity, and it is the most common resistance mechanism described till now in various Gram negative bacteria^[34]^. Mutations at codon Ser83 or/and Asp87 in quinolone resistance determining region of *gyrA* are the two most common sites leading to resistance in quinolone^[35]^. Previous studies have indicated that in *Salmonella* the most common residues associated with mutation are at serine 83 and aspartate 87, either alone or together.^[35]^ This study described the mutation in gyrase gene which has been clustered in a QRDR due to observed resistance for quinolone (nalidixic acid) group of antibiotic. Giraud *et al*.^[35]^ reported that a single point mutation in *gyrA* gene of *Salmonella* is adequate to cause high level of quinolone resistance, the same was observed in the present study. A single point mutation of strain S130 at position 87 in the quinolone resistance determining region, caused substitution in amino acid from aspartic acid to glycine. This showed reduced susceptibility to ciprofloxacin (MIC 0.032 µg/mL). In the study, some silent mutations were observed at different points which make no changes in the protein or enzyme (gyrase A) conformation.

Apart from development of quinolone resistance by mutation Martinez *et al*.^[13]^ recognized the plasmid mediated quinolone resistance determinant, qnr protein (*qnrA1*) on a conjugative plasmid isolated from ciprofloxacin resistant *Klebsiella pneumoniae.* Similarly, the isolates from the present study harbored *qnr* genes in their plasmid provided evidence that the plasmid mediated resistance transfer and it is in agreement with Hernandez *et al*.^[35]^. The results from the study were corroborated with earlier reports and 26 (72%) of 36 isolates carried at least one or more *qnr* genes in their plasmid which is an accountable for resistance to nalidixic acid and reduction in susceptibility against ciprofloxacin. Among 36 isolates, 15 (42%) carried *qnrA*, 11 (30%) carried *qnrB* and 26 (72%) carried *qnrS* genes. This demonstrated that the plasmid mediated quinolone resistance can be transferred between *Salmonella* populations from different sources.

In conclusion, the occurrence of high rate of antimicrobial resistance among NTS strains isolated from food of animal origin has emerged as a serious problem in India. Our findings in the study clearly demonstrated the acquirement of multi-drug resistance in NTS isolated from poultry and seafood. Besides, interestingly, the first line antibiotics showed their antimicrobial activity against multi-drug resistant NTS. Since the antibiotic resistant determinants were found on the plasmid (mobile genetic element), there is a strong chance for transmission of resistance among bacterial population. This study clearly demonstrates the need for regular monitoring of antibiotic use and an effective antibiotic policy to prevent the indiscriminate use of antibiotics in different fields.
